# Secondary school practitioners’ beliefs about risk factors for school attendance problems: a qualitative study

**DOI:** 10.1080/13632752.2019.1647684

**Published:** 2019-08-01

**Authors:** Katie Finning, Polly Waite, Kate Harvey, Darren Moore, Becky Davis, Tamsin Ford

**Affiliations:** a College of Medicine and Health, University of Exeter, Exeter, UK; b School of Psychology & Clinical Language Sciences, University of Reading, Reading, UK; c Graduate School of Education, University of Exeter, Exeter, UK

**Keywords:** School attendance, school absence, teachers, school mental health, qualitative

## Abstract

School staff have an important role to play in identifying and assisting pupils who require additional support to regularly attend school, but their beliefs about risk factors might influence their decisions regarding intervention. This qualitative study investigated educational practitioners’ beliefs about risk factors for attendance problems. Sixteen practitioners from three secondary schools were interviewed via focus groups. Data were analysed using thematic analysis. Practitioners identified risk factors related to the individual, their family, peers and school. Poor mental health was identified as a risk factor, but practitioners primarily focused on anxiety rather than other mental health problems like depression or behavioural disorders. The overall perception was that school factors were less important than individual, family and peer factors. Implications include a need for increased awareness of the role of school factors in attendance problems, focus on promoting positive peer and pupil-teacher relationships, and collaborative working between young people, families and schools.

## Introduction

School plays a crucial role in young people’s academic, emotional and social development, and frequent absence from school is associated with a range of adverse consequences both in the short- and long-term, including poor academic outcomes (Credé, Roch, and Kieszczynka 2010), economic deprivation (Kearney 2008b), and adult unemployment (Attwood and Croll 2014). In the 2017/18 academic year, 8.7% of primary and 13.9% of secondary school pupils met criteria for persistent absence, defined by the Department for Education as missing 10% or more of available school sessions (Department for Education 2019). Furthermore, rates of authorised, unauthorised and persistent absence have increased in the last year, and unauthorised absences are now the highest since records began (Department for Education 2019).

Previous researchers have commonly divided risk factors for attendance problems into those related to the individual, their family, school, and peers (Gren-Landell et al. 2015; Ingul et al. 2012; Ingul, Havik, and Heyne 2019), in line with Bronfenbrenner’s ecological theory, which recognises the important role of factors in all of these domains in child development (Bronfenbrenner and Morris 2006). In terms of factors related to the individual, research has demonstrated that poor physical health (Ingul et al. 2012), mental health problems (Egger, Costello, and Angold 2003), special educational needs (Havik, Bru, and Ertesvåg 2015b) and drug or alcohol use (Gase et al. 2014) are risk factors for poor attendance. Factors related to the family include neglectful parenting (Gase et al. 2014), lack of parental involvement in school activities (Hendron and Kearney 2016), unemployment (Ingul et al. 2012), family conflict (McShane, Walter, and Rey 2001) and family history of attendance problems (Dalziel and Henthorne 2005). School factors include poor school climate (including connectedness and perceptions of school safety (Van Eck et al. 2017)), poor pupil-teacher relationships (Egger, Costello, and Angold 2003; Malcolm et al. 2003) and school transition periods (Malcolm et al. 2003). Finally, factors related to peers include social isolation (Havik, Bru, and Ertesvåg 2015a), a lack of peer support (Hendron and Kearney 2016), peer conflict (McShane, Walter, and Rey 2001), bullying (Ingul et al. 2012) and pressure from peers to skip school (Malcolm et al. 2003).

Despite the common categorisation of risk factors into these four broad domains, in reality, complex interplays exist within and between these categories, as reflected by ‘mesosystems’ in Bronfenbrenner’s ecological theory (Bronfenbrenner and Morris 2006). For example, poor pupil-teacher relationships and bullying can both negatively impact mental health (Lang et al. 2013; Barchia and Bussey 2010), and parents with a history of attendance problems may be less inclined to involve themselves in their child’s school activities. In addition, some risk factors could be argued to fall under multiple domains. For example, a lack of parental involvement in school activities is often considered a family factor, but may also be influenced by the young person (individual factors) and/or the school environment (school factors). Whilst grouping risk factors into broad domains may be helpful for conceptualisation of the problem, in practice it is likely that attendance problems result from complex interactions between risk factors, and the best approaches are likely to involve interdisciplinary collaboration between professionals in the fields of education and healthcare, as well as between these professionals and families (Heyne 2019; Kearney 2008a; Gren-Landell et al. 2015).

A further complicating factor in school attendance research is the continued debate regarding terminology. A full discussion is outside the scope of this paper, but a brief description is provided and interested readers are directed towards more in-depth discussions provided elsewhere (e.g. (Elliott and Place 2017; Heyne et al. 2018; Kearney 2008b). School attendance has historically been divided into two subtypes: (a) school refusal, referring to pupils who miss school due to anxiety or emotional distress, with the knowledge of their parents; and (b) truancy, referring to pupils who miss school due to a lack of interest in school or defiance of authority, and who attempt to conceal the absence from their parents. School refusal is typically considered to be related to internalising problems such as depression or anxiety, while truancy is considered to be related to externalising problems. However, a study by Egger, Costello, and Angold (2003) demonstrated that school refusal and truancy are not mutually exclusive, and other studies, including two linked systematic reviews, have shown that truancy is strongly associated with internalising problems (Finning et al. 2019a, 2019b; Mandalia et al. 2018; Gase et al. 2014). It could therefore be argued that grouping attendance problems into subtypes such as school refusal and truancy lacks empirical support, and may result in adults around the young person making inaccurate assumptions about the underlying aetiology of the problem. Indeed, truancy is viewed less sympathetically by school staff than school refusal and is more likely to be approached punitively rather than therapeutically (Armstrong et al. 2011).

School staff are likely to be among the first to recognise poor or changing patterns of attendance and play a central role in identifying pupils who are struggling to attend. It is important, therefore, to understand the beliefs of school staff regarding risk factors for attendance problems. In a survey of Swedish teachers, family factors and child low mood/depression were believed to be the two leading causes of attendance problems in young people aged 12 to 15 years (Gren-Landell et al. 2015). In a quantitative survey in the UK, Malcolm et al. (2003) found that primary and secondary school teachers believed home factors such as inadequate parenting, a disorganised lifestyle, and low value placed on education, to be causes of truancy. Secondary school teachers additionally discussed the influence of non-familial factors such as bullying, pressure from peers to miss school, a curriculum not suited to the pupil’s needs (e.g. over-academic or ‘boring’), and school change or transition.

The majority of previous research has investigated teachers’ views while overlooking the experiences of other school staff who play an important role in identifying and responding to attendance problems, including those with greater pastoral roles. A qualitative study by Cunningham (2017) explored the experiences of primary school practitioners in a variety of teaching and non-teaching roles. Practitioners in this study discussed a range of factors they believed increased pupils’ risk of attendance problems, including anxiety, low academic confidence, peer difficulties, low family aspirations, parental anxiety and overprotection, family deprivation, and a chaotic home life. However, factors related to the school were rarely discussed, supporting findings from previous research that teachers perceive home-life as the primary cause of attendance problems, despite pupils and parents emphasising school factors (Gregory and Purcell 2014; Havik, Bru, and Ertesvåg 2014; Malcolm et al. 2003; Gren-Landell et al. 2015; Dannow, Esbjørn, and Risom 2018).

To the best of our knowledge there have been no qualitative studies to explore secondary school educational practitioners’ beliefs about risk factors for attendance problems. Given that rates of overall and persistent absence are higher in secondary, compared to primary, school (Department for Education 2019), it is important to understand attendance problems from the perspective of those who have experience working with this age-group. This study aims to investigate secondary school educational practitioners’ beliefs about risk factors for school attendance problems.

## Methods

Data were collected via focus groups, which are useful in generating a rich understanding of experiences and encouraging participants to make collective sense of phenomena. We used focus groups rather than individual interviews as the former more readily highlights similarities and differences between individual views, allows group members to challenge each other’s opinions, and may generate a wider range of views and ideas than could be captured through individual interviews (Barbour 2007; Kidd and Parshall 2000; Morgan 1998).

### Sample

Opportunity sampling was used to recruit 16 secondary school educational practitioners from three schools in the South West of the UK, with one focus group conducted at each of the three schools. Table 1 provides further details of the three schools. Practitioners could be working in any teaching or non-teaching role, but were required to have experience of working with pupils with attendance problems. Table 2 provides further details of the 16 practitioners who participated.

**Table 1.** Characteristics of participating schools.

Focus group	School type	Ofsted inspection rating	Total number of pupils	Pupils eligible for Free School Meals (%)	Rate of overall absence (%)	Rate of persistent absence^a^ (%)
1	Mainstream city-centre faith academy ^b^	3 – Requires improvement	1646	9.4	7.1	21.9
2	Mainstream rural academy	2 – Good	1518	4.5	5.1	11.8
3	Mainstream city-centre academy	2 – Good	908	11.5	6.9	20.4

Source: DfE school comparison tool via www.compare-school-performance.service.gov.uk. Data refers to the 2016/17 school year.

^a^Persistent absence refers to the percentage of pupils who miss 10% or more of school sessions in a year; National average is 13.5%.

^b^Academies are independent, state-funded schools that receive funding directly from central government, and are independent of local authority control.

**Table 2.** Participant characteristics.

Participant	Focus group	Gender	Age	Job role
P1	1	Male	40–49	Head of Key Stage Four*
P2	1	Male	30–39	SENCO
P3	1	Male	40–49	Assistant Head of Sixth Form
P4	1	Female	30–39	Head of Year
P5	1	Male	30–39	Head of Year & P.E. teacher
P6	1	Female	40–49	Head of Year 9
P7	2	Female	40–49	SENCO
P8	2	Male	50–59	Assistant Principal
P9	2	Female	30–39	Parent & Family Support Advisor
P10	2	Female	20–29	Pupil Support Worker
P11	2	Female	60+	Inclusion Manager
P12	2	Female	40–49	Pupil Support Worker
P13	3	Female	50–59	Family Liaison Worker
P14	3	Female	40–49	Personalised Learning Assistant
P15	3	Female	40–49	Personalised Learning Assistant
P16	3	Female	30–39	Deputy Safeguarding Lead

*Key Stage Four refers to school Years 10 and 11, when pupils are aged between 14 and 16 years. P.E. = Physical Education; SENCO = Special Educational Needs Coordinator.

### Data collection

Focus groups were conducted as part of a broader project that aimed to explore practitioners’ experiences of working with pupils with attendance problems, and the interventions available. A semi-structured topic guide was used to encourage consistency in the topics covered, while also allowing flexibility for practitioners to discuss topics pertinent to their own experience. The topic guide included questions regarding practitioners’ experience of working with pupils with attendance problems, the current support available, and further support they believed would be beneficial. The term ‘attendance problems’ is used throughout this paper to reflect the broad nature of focus group discussions. Findings from our initial analysis exploring general experiences and interventions for attendance problems have been published previously (Finning et al. 2017). However, during the original analysis, we noticed that data had been collected from focus groups that focused on practitioners’ beliefs about risk factors for attendance problems. This topic was not intended according to our original research aims, nor in the topic guide, but was clearly viewed as important to participants. This additional topic is therefore the focus of the research question and analysis presented in this paper. Secondary analysis of qualitative data in this way is not uncommon and enables deeper understanding of particular issues arising in the data (Wästerfors, Åkerström, and Jacobsson 2014). Ethical approval was provided by the University of Reading Research Ethics Committee.

### Procedure

Eighteen schools were initially approached, via an email from BD, which was followed up with a phone-call. Three schools agreed to participate. Practitioners from these schools were recruited to the study via word-of-mouth from a lead point of contact at the school. Focus groups were conducted within school grounds, during or at the end of the school day, by BD who had prior experience as a teacher and was undertaking an MSc in Psychology. Each focus group was additionally attended by a moderator, who assisted BD and took notes. Practitioners had no relationship with either researcher prior to their participation in the study. Written consent was provided by all practitioners. Focus groups lasted between 39 and 54 minutes, and were audio-recorded, transcribed and double-checked for accuracy by KF.

### Analysis

Thematic analysis, as described by Braun and Clarke (2006), was used to analyse data, with the assistance of QSR International’s NVivo 11 software. Thematic analysis is a flexible technique that aims to identify, analyse and organise patterns within the data. ‘Codes’ are labels that are applied to the data according to their meaning, and these are then organised into higher-level ‘themes’ (Braun and Clarke 2006). Transcripts were initially read and re-read by KF by way of familiarisation with the data, and then codes were applied to the transcripts line-by-line. We used a combination of deductive coding, based on our knowledge of previous literature, and inductive coding of topics that were identified during analysis. The coded data were grouped into themes and subthemes based on their semantic similarity. Coded data were then reviewed to ensure that themes and subthemes were coherent and distinct. Finally, transcripts were re-read to relate the themes back to the original data, and to provide an opportunity for any final coding to take place. Although this process is described linearly, it was in fact an iterative and cyclical process, which continued until a final map of themes was produced. Throughout analysis, meetings were held between KF, PW and KH, in order to discuss emerging codes and themes. Data within each theme were summarised in order to produce a narrative, which is presented in this paper.

## Results

**Figure 1.**
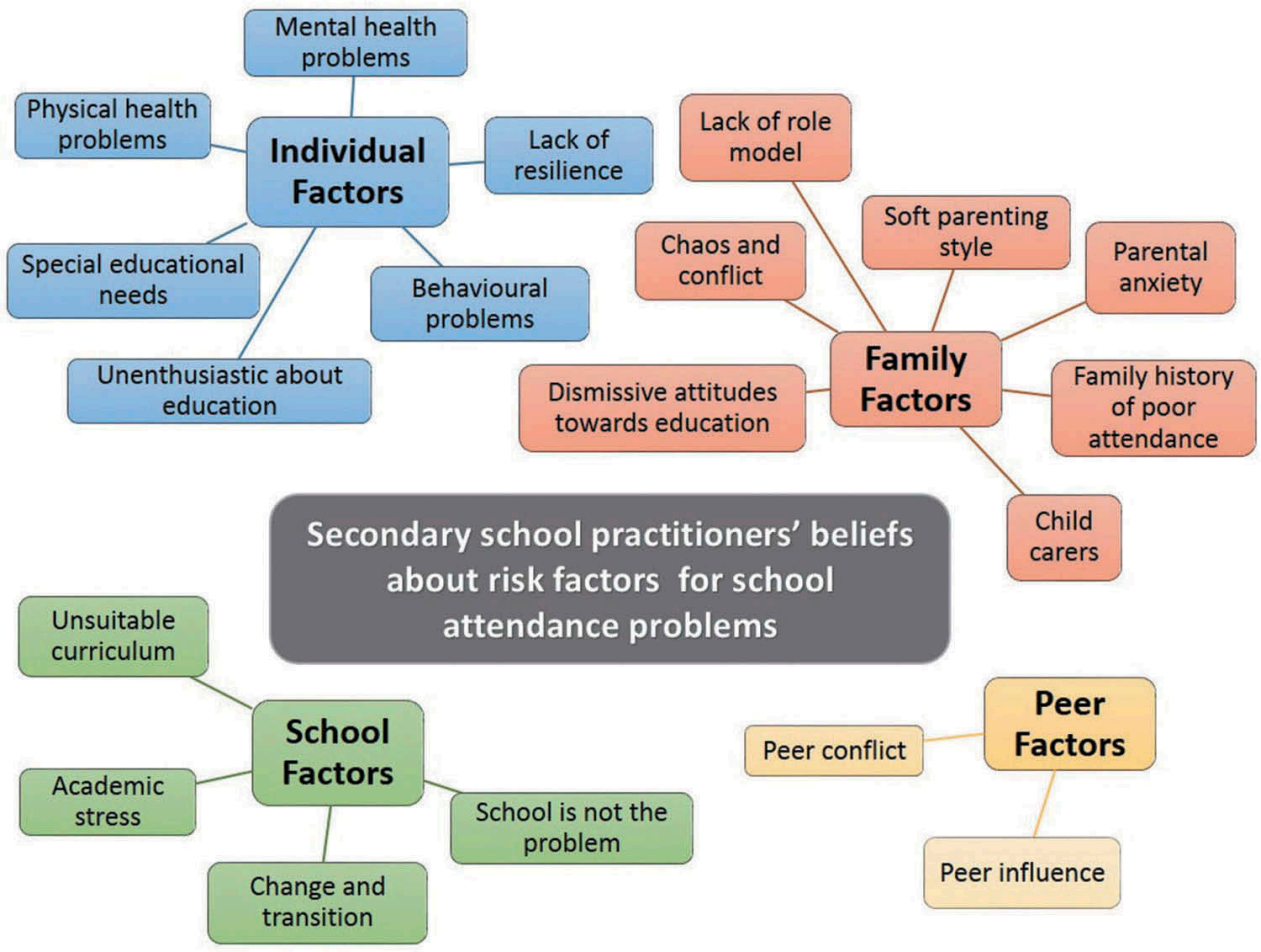
Analytic themes and factors identified within each theme.

Analysis identified four major themes, related to individual, family, peer, and school factors. Figure 1 shows the four themes and the factors identified within each theme.

### Individual factors

Practitioners in all three groups expressed a belief that mental health problems, particularly anxiety, are a factor in the majority of cases of attendance problems, and that although missing school reduces anxiety in the short-term, it ultimately causes pupils’ anxiety to build, thus creating a vicious cycle.

I do think that the majority that we’ve worked with have all been something to do with mental health issues, and it starts with a simple panic attack at school and then it escalates until it’s full-blown school anxiety. And once they’re there, it’s very difficult to get them back in again. (P6, Head of Year Nine, Group One)

Mental health problems were considered a particular risk factor for secondary school pupils rather than younger children, and were highlighted as an issue that may be increasing. Anxiety was seen to fluctuate, which could make it difficult to identify:

The anxiety might manifest itself with a particular subject or particular teacher or particular friendship group, but that wouldn’t be there in another lesson or at lunch-time … so that’s really hard to see that change. (P8, Assistant Principal, Group Two)

All three groups identified a lack of resilience as a key risk factor for attendance problems. Practitioners described pupils with difficult life circumstances who maintained regular attendance, which was believed to be a result of resilience. Pupils with a lack of resilience were described as being sensitive to seemingly small triggers such as minor peer conflict.

The kids that are strong, they’ve had a bit of conflict and come back the next day. (P9, Parent and Family Support Advisor, Group Two)

Groups One and Two described a perceived dichotomy between mental health and bad behaviour, and discussed pupils they believed had poor attendance because they were ‘*rebelling’ (P6, Head of Year Nine, Group Two)* or being a ‘*naughty kid’ (P1, Head of Key Stage Four, Group One)*. Although such behaviours may be a sign of mental or neurodevelopmental disorders, practitioners appeared not to label these behaviours as mental health problems, and implied the use of tougher, more punitive approaches towards these pupils compared to those believed to have internalising problems such as anxiety.

You think well I’m just rewarding bad behaviour and if you do that you’re just opening the flood gates, and that’s always the fine line that you’re always walking, with so many kids. (P1, Head of Key Stage Four, Group One)

A lack of enthusiasm towards school and low academic aspirations were considered risk factors, and practitioners discussed pupils who they believed simply *‘can’t be bothered’ (P4, Head of Year, Group One)* to attend school, or prefer to do other things such as hang out with friends or go to the park.

We used to have a girl like that who used to get on the bus and go to [name of park] for the day (laughs). (P14, Personalised Learning Assistant, Group One)

Used to have a lovely day. (P15, Personalised Learning Assistant, Group One)

Groups One and Two identified special educational needs as a risk factor for poor attendance, believing that mainstream school is not suitable for everyone and that for pupils who are struggling to attend school, alternative schooling should be considered.

Mainstream school’s not for everyone, we’re going to have that small group of kids that no matter how hard you try, school is just not the right place for them, they need something different. (P6, Head of Year Nine, Group One)

Only Group One acknowledged the role of physical health problems, although they did not discuss this at any great length and one practitioner was unconvinced of the validity of some pupils’ medical absences:

You get these medical reasons but then you sort of think yeah, but I think if push had come to shove, that pupil could have been in more. (P1, Head of Key Stage Four, Group One).

### Family factors

Family life was emphasised as playing a key role in attendance problems. Practitioners described parents with dismissive attitudes towards education and low aspirations for the family, often as a result of their own upbringing or cultural norms, and some parents were perceived to support non-attendance and be obstructive towards the school’s attempts to engage the pupil.

Your biggest problem with school refusers is home sadly, and I hate to put it back onto the parents … but the majority of cases, the parents aren’t backing us, they might say that they are while on the phone but they’re not backing us up. (P1, Head of Key Stage Four, Group One)

Group One highlighted the negative impact of chaos or conflict within the family home:

She’s been better the past few weeks, there was today but that seemed to be a real conflict at home that’s kinda, hard to resolve. (P3, Assistant Head of Sixth Form, Group One)

Her and mum just don’t get on. (P6, Head of Year Nine, Group One)

On the other hand, practitioners described the positive impact of parents who consistently encourage their child to attend school even when they are struggling:

They’d come to me in the morning even if they were upset, so they knew that their parent would still bring them in and they wouldn’t, whereas if you had a parent that was like ‘oh well they’re not coping, they can’t do it’ … (P10, Pupil Support Worker, Group Two)

Yes then that could have developed into a different story. (P9, Parent & Family Support Advisor, Group Two)

Practitioners discussed the detrimental effect of a lack of positive role model at home, such as parents who are *‘still in their pyjamas or not even up … .then the child’s got no motivation to get themselves out of bed to get an education’ (P15, Personalised Learning Assistant, Group Three)*. Also perceived to be detrimental to attendance was a ‘soft’ parenting style such as not following through with consequences, a lack of boundaries, or prioritising avoidance of conflict over attendance. Parental anxiety was also considered a risk factor, making it difficult to engage parents and creating a barrier for their child to attend. As P11 explained:

‘*The anxious parents that you know, their kid wants to come to school but the parent’s too anxious and that’s really difficult.’ (P11, Inclusion Manager, Group Two)*

Attendance problems were considered to sometimes be a transgenerational issue; a *‘trait’ (P4, Head of Year, Group One)* that runs through families, often having previously occurred with siblings, parents or grandparents. Practitioners implied that parental school anxiety and/or attitudes towards education were important factors in this respect:

And it runs in families as well doesn’t it sometimes? (P11, Inclusion Manager, Group Two)

I was just going to say that, I don’t know whether your research will look at sort of generational impact as well, you know the, the ethos or the mind-set of that family unit, you know, were parents, were grandparents attenders at school, were they successful at school, um, that has a huge bearing doesn’t it? (P8, Assistant Principal, Group Two)

Two groups discussed the challenges of attendance for pupils with caring responsibilities at home, for example if there is parental illness or domestic abuse:

If parents have been through a traumatic time they don’t want to leave their parent … had one young lady that was probably caring for her younger siblings, um, and her mum had been subjected to some unpleasantness from her partner so it was easier to stay at home, protect mum and the siblings. (P14, Personalised Learning Assistant, Group Three)

### Peer factors

Only two factors related to peers were identified by practitioners: peer conflict and negative peer influence. Peer conflict was considered a risk factor for attendance problems, especially for girls. In particular, Group One discussed the role of social media, which they believed prevents some pupils being able to escape from difficult social relationships. Peer conflict was considered to have the potential to cause sudden and severe attendance problems in pupils who previously had good attendance.

It could have been something as tiny as a little bit of, maybe, verbal bullying you know, bit of name-calling, that they would have gone home and dwelled on it, or it could be a really serious bullying. (P15, Personalised Learning Assistant, Group Three)

Practitioners in Group One also described the role of negative peer influence, for example when pupils were considered to *‘get in with a bad group of kids’ (P6, Head of Year Nine, Group One)*, or when a pupil with poor attendance caused classmates to question their own decision to attend:

There is a danger of it impacting on others as well isn’t it because I used to have [pupil’s name] and, and actually someone in the class would say ‘well actually she’s never in’ and, you know, and ‘why bother?’, and they start questioning it as well. (P2, Special Educational Needs Co-ordinator, Group One)

### School factors

Group One described the UK National Curriculum as unsuitable for some pupils and believed that offering more vocational subjects, as well as a curriculum that could be tailored to individual needs, would improve attendance. However, the group agreed that this would be difficult, as P2 explained: *‘A lot of schools aren’t able to offer the appropriate curriculum anymore because of the cost of, and the pressures and expectations’ (P2, Special Educational Needs Co-ordinator, Group One).*

Practitioners in Group Three identified academic stress, particularly during exam periods, as a potential cause of attendance problems. This was considered especially influential when combined with other life stressors:

I think the pressure, um, on achievement in the older years can be massive … they’ve got all these pressures going on amongst the family, or you know, outside and so on and then you add on GCSEs. (P16, Deputy Safeguarding Lead, Group Three)

Times of change and transition were considered to be high-risk for the onset of attendance problems, including returning to school after the summer holidays, changing schools mid-term, and, in particular, the transition from primary to secondary school:

The difference is they have one teacher most of the time, you come here and they may have 15 different teachers in a week and those, not only the transition from primary to senior but transition to every hour of the day, across a big school can be just so mind blowing. (P11, Inclusion Manager, Group Two)

Although some school factors were identified, they were discussed less frequently than factors related to the individual, their family and their peers. Group One, in particular, did not discuss any school factors except for the Curriculum. Although previous studies have also reported that school staff de-emphasise the role of school factors (Dannow, Esbjørn, and Risom 2018; Gregory and Purcell 2014; Gren-Landell et al. 2015; Malcolm et al. 2003) practitioners in this study went one step further, with Groups One and Three concluding that they do not believe school contributes to the problem for the majority of pupils:

When it boils down to it it’s not school related is it? (P14, Personalised Learning Assistant, Group Three)

No because if you removed all the barriers that they say are the issue with school, then you’d still have the same problems … So yes it’s everything that happens outside school. (P16, Deputy Safeguarding Lead, Group Three)

It’s outside of school isn’t it? (P1, Head of Key Stage Four, Group One)

Yes, it’s not anything we are doing. (P6, *Head of Year Nine, Group One)*

Group Two, whilst not explicitly denying a role for school, did suggest that as young people spend most of their time away from school, the majority of influence comes from other sources:

Whilst we have continuing, consistency of them coming into school for five days perhaps, it is only 25% of their day isn’t it, and 75% they’re with others, so the influence we have is, is restricted, it’s a big chunk of time but … there’s a much bigger chunk of time outside of school hours. (P8, Assistant Principal, Group Two)

## Discussion

This qualitative study explored secondary school educational practitioners’ beliefs about risk factors for school attendance problems, which was identified as an important topic during a study that investigated practitioners’ broader experiences of attendance problems. Practitioners identified a range of factors they believed to be associated with poor attendance, which were grouped into those related to the individual, their family, their peers, and the school. We used these four broad groups of risk factors for the purposes of analysis because this grouping has commonly been utilised in previous research (Gren-Landell et al. 2015; Ingul et al. 2012; Ingul, Havik, and Heyne 2019), and because these four domains of risk factors seemed to be endorsed by practitioners in this study. However, it is important to highlight that risk factors from each domain are unlikely to stand alone, and the potential for interaction between factors will be considered later in this discussion.

Factors related to the individual that were identified by practitioners in this study included mental health problems (particularly anxiety), a lack of resilience, behavioural problems, poor engagement with education, special educational needs, and physical health problems. The focus by practitioners in this study on anxiety as opposed to other mental health problems is interesting given that quantitative studies demonstrate depression to be an even greater risk factor (Egger, Costello, and Angold 2003; Finning et al. 2019a). Although behavioural difficulties were identified as a risk factor, practitioners were likely to label this as ‘naughty’ or rebellious behaviour rather than a symptom of mental ill health, which has important implications for pupils given that previous research has shown that teachers use such labelling to drive their decisions over who needs support as opposed to punitive intervention (Torrens Armstrong et al. 2011).

Family factors identified included dismissive attitudes towards education, chaos or conflict at home, a lack of positive role model, a ‘soft’ parenting style, parental anxiety, and young people with caring responsibilities. Similarly, previous research has demonstrated that family factors such as neglectful parenting, parental ill health, a lack of parental interest, and family conflict are associated with poor attendance (Gase et al. 2014; Hendron and Kearney 2016; McShane, Walter, and Rey 2001). Practitioners in this study identified family history of attendance problems as a risk factor, which is supported by a UK survey that reported 26% of children whose parents had poor attendance missed school for reasons other than illness, compared to 10% of children whose parents had good attendance (Dalziel and Henthorne 2005).

Practitioners identified two risk factors related to peers: peer conflict, and peer influence, for example when pupils were considered to make friends with a ‘bad crowd’ who encouraged them to skip school. Previous research has demonstrated that peer conflict and bullying negatively impact on attendance and on mental health (Ingul et al. 2012; McShane, Walter, and Rey 2001), although practitioners in this study believed that even mild conflict could result in attendance problems. The reference by one practitioner in this study to *‘something as tiny as a little bit of … verbal bullying’ (P15, Personalised Learning Assistant, Group Three)* aligns with previous research that has demonstrated school staff consider non-physical forms of bullying to be less severe than physical bullying (Hazler et al. 2001). Finally, practitioners discussed the role of school factors in attendance problems and identified academic stress, an unsuitable curriculum (lack of vocational subjects; inability to tailor to individual pupils’ needs) and school change or transition, as potential factors. Previous research shows that parents also believe academic stress and pressure, particularly around exams, is a cause of attendance problems (Dalziel and Henthorne 2005). It is notable that overall, despite identifying several school factors, these were discussed less frequently than other factors, which supports findings from previous studies that teachers perceive individual and family factors to be the primary cause of attendance problems, while pupils and parents emphasise school factors (Dannow, Esbjørn, and Risom 2018; Gregory and Purcell 2014; Gren-Landell et al. 2015; Havik, Bru, and Ertesvåg 2014; Malcolm et al. 2003). Despite acknowledging some school-related risk factors, practitioners also stated that they do not believe school to be the cause of attendance problems. This is important given that a range of school factors are associated with poor attendance (Egger, Costello, and Angold 2003; Malcolm et al. 2003; Van Eck et al. 2017), and positive school factors such as supportive pupil-teacher relationships can reduce the impact of other stressors on negative educational outcomes (Hamre and Pianta 2005). In addition, parents believe that supportive school staff are crucial for re-engaging pupils with poor attendance (Havik, Bru, and Ertesvåg 2014).

Taken together, our findings suggest a perceived lack of agency by school practitioners in terms of their ability to influence risk factors for attendance problems. For example, practitioners recognised academic stress as a risk factor. Previous studies have shown that teachers contribute to academic stress, particularly leading up to GCSEs (Putwain 2011), but practitioners in this study did not acknowledge their potential role in contributing to, or being able to help mitigate, pupils’ stress. Practitioners also recognised peer conflict and bullying as a risk factor but, again, did not discuss the role that they, either as individuals or at a school-level, might play in attenuating this risk, for example through emphasising a positive school culture or implementation of anti-bullying policies (Davies 2013). School-based issues are those that school staff are likely to have some influence over and it is important that school staff are encouraged to consider the ways in which they may be able to exert positive influence on attendance problems (Moore et al. Under Review). Small, positive shifts in some of the things that school practitioners can control could be the difference between attendance and non-attendance, particularly for pupils who are experiencing other life stressors. The framework presented here, which separated risk factors into those related to the individual, their family, school and peers, is a commonly utilised framework in the school attendance literature (Gren-Landell et al. 2015; Ingul et al. 2012; Ingul, Havik, and Heyne 2019). Practitioners in this study appeared to endorse these categories, particularly given their emphasis on the importance of risk factors in some domains but not others. In reality, however, complex interplays exist between the young person, their friends, home-life and school, as reflected by mesosystems in Bronfenbrenner’s ecological model (Bronfenbrenner and Morris 2006). At times, the interplay between different domains was recognised by practitioners in this study. For example, practitioners believed that differences in resilience (individual factor) determines whether pupils who are experiencing peer conflict (peer factor) or difficulties at home (family factor) struggle to attend school, or manage to maintain good attendance. Practitioners in Group Three also discussed how the pressure of GCSEs (school factor) can be more problematic if the pupil also has pressures at home (family factor).

Risk factors for attendance problems are unlikely to occur in isolation, and successful intervention may require consideration of the interaction between risk factors within each domain (e.g. how mental health and physical health interact) and across domains (e.g. how teacher-pupil relationships are influenced by parental attitudes and experiences). Collaboration between the young person and adults across all of these domains is likely to be key for successful intervention (Gren-Landell et al. 2015; Heyne 2019; Kearney 2008a).

Given that risk factors for attendance problems were not included as a probe in the topic guide, it is notable that not only did all three groups spontaneously discuss this topic, but there was also a large degree of consistency both within and between the three groups in terms of the risk factors identified. In fact, while there were some subthemes that were only discussed by one or two of the groups, there were no instances where groups expressed opposing views. This is interesting given that there was variation both in terms of the three schools included in the study and in terms of the experience and job roles of individual practitioners. For example, participants included teachers, members of senior leadership, SENCOs and support staff. This finding confirms that risk factors are common across the schools included in this study, and salient to practitioners working in a variety of roles.

### Strengths and limitations

This study was reported in accordance with best practice guidelines for the reporting of qualitative studies (Tong, Sainsbury, and Craig 2007). We interviewed practitioners in a variety of teaching and non-teaching roles, in order to gain diversity of experience. The three schools from which practitioners were sampled were also diverse in terms of their setting, size and absence rates. However, our opportunity sampling method was likely to have reduced diversity in other respects. All practitioners worked in mainstream state-funded academies, and all had experience of working with pupils with attendance problems and are likely to be interested in this topic area. Practitioners working in special education settings, and those less engaged with attendance problems may hold different views, which were not explored in this study. Practitioners in a variety of roles and with different levels of experience were interviewed in focus groups together, and it is possible that these differences may have prevented some practitioners from openly expressing their views. Data saturation was not formally assessed, and it is possible that additional themes may have been identified if further focus groups had been conducted. The option of conducting additional focus groups was discussed throughout the process of analysis, but was considered unnecessary as the data obtained was sufficiently rich in order for us to address our research aims.

### Implications

Our findings suggest that secondary school practitioners are aware of many of the most common causes of attendance problems, but in general factors related to the individual and their family were highlighted, while school factors were de-emphasised. School factors are likely to be among those over which practitioners have the greatest control and it is important that school staff are encouraged to consider the role of school factors and their ability to create change for pupils with attendance problems (Moore et al. Under Review). Given that peer conflict and bullying are identified as risk factors, it is essential that schools implement anti-bullying policies, supplemented with the use of evidence-based bullying interventions where necessary (Davies 2013). Schools should take steps to encourage pupils to develop healthy relationships with peers and engage in positive activities, for example through peer mentoring schemes or links to voluntary sector activities.

Academic stress was also recognised as a risk factor for attendance problems, particularly when combined with other life stressors. Following the UK Government’s changes to GCSE examinations in 2017, there have been widespread reports of increased stress and declining mental health in secondary school pupils (e.g. Weale (2018)), although the impact of this has yet to be formally investigated. Findings presented here and in a previous study suggest that such stress may not only be harmful to pupils’ mental health, but that it may also negatively impact attendance (Dalziel and Henthorne 2005). Schools may be able to minimise the impact of exam stress by providing additional support, for example through the provision of skills-based training such as study skills or time management.

Practitioners identified mental health problems as a risk factor for school attendance, but largely focused on the influence of anxiety while neglecting to discuss the impact of other mental health problems such as depression or behavioural disorders. Indeed, behavioural difficulties were discussed in the context of ‘naughty’ or ‘rebellious’ pupils, without acknowledgement that such behaviour may in fact be a sign of a mental health problem. Research has demonstrated that depression is an even greater risk factor for attendance problems than anxiety (Egger, Costello, and Angold 2003; Finning et al. 2019a), and schools need to be aware that poor attendance may be a sign of a range of mental health difficulties, not only anxiety.

Practitioners identified that pupils with caring responsibilities may be at increased risk of attendance problems, for example when parents or siblings have mental or physical health problems, or when there is abuse in the home. Developing strong school-family relationships may help to improve schools’ knowledge of individual family circumstances, and enable them to offer additional support and flexibility for these pupils and signpost families to appropriate support services.

Practitioners believed that resilience is a key factor that distinguishes pupils who, despite experiencing adversity, maintain good attendance, from those who are frequently absent. Schools may be able to support pupils in developing personal resilience, for example through Personal, Social, Health and Economic (PSHE) lessons in the UK (PSHE Association 2017). Positive relationships with caring adults and peers, and effective teachers and schools are identified as key correlates of resilience in young people (Sapienza and Masten 2011). Therefore, school environments that promote positive relationships between pupils, and between pupils and staff, are likely to promote resilience and have the potential to positively impact pupils’ health and educational outcomes.

## Conclusion

This study identifies a range of risk factors that secondary school educational practitioners believe contribute to school attendance problems. These include some school factors, but the perception was that these factors were less important than individual, family and peer factors. Practitioners recognised the influence of mental health on attendance, but focused on anxiety rather than depression, behavioural disorders or other mental health problems. Given that school staff are best placed to directly influence factors related to the school, we suggest a number of steps that school staff can take that may be beneficial. This includes implementation of anti-bullying policies, promoting positive peer and pupil-teacher relationships, and collaborative working between young people, families and schools.
