# Supplementary material for: Secondary school practitioners’ beliefs about risk factors for school attendance problems: a qualitative study
Source: Emot Behav Diffic. 2019 Aug 1;25(1):15–28. doi: 10.1080/13632752.2019.1647684 (PMC7254525; doi:10.1080/13632752.2019.1647684)
Supplement: Supplemental Material [file REBD_A_1647684_SM5651.docx]

## Focus group topic guide

### Discussion topic 1 – Previous experience of school refusal

• What have been the challenges of having a student who is having difficulty attending school?

• When you have had a student with these difficulties, what was the pattern of their attendance beforehand (i.e. did their attendance gradually get worse or was it more sudden; was it predictable or not)?

### Discussion topic 2 – The role of teachers

Now consider the role staff in supporting students who display signs of school refusal.

• When you have been involved with a student who isn’t coming into school, what sort of things have you been doing??

• Can you describe instances when school refusal students have responded positively to what you or other teachers have done? How did the students’ behaviour change? What approach did you take?

• Are there any times when what the teacher has done might have had a negative impact? Can you give examples without disclosing personal details?

• Do you think data is important when dealing with school refusal? (Question was added for final focus group)

### Discussion topic 3 – The support available

Think about the support and intervention that is available in this school.

• When there have been students who are not attending school, what sort of support have they received?

Describe/give details?

• What about when they have been off school for a long time – months or years?

• What support strategies have been successful? Why do you think they were successful?

• How important has it been to involve parents?

• Are there any strategies have been unsuccessful? Why do you think they were unsuccessful?

• Have the teachers (either individually or as a school) received any training or support?

### Discussion topic 4 – Further support for school refusal

Consider specific gaps in the support for school refusal students, their families and school staff.

• Are there any changes would you make to the support and intervention that is currently available? Please give details.

• Imagine you had unlimited time and resources, in an ideal world what support and interventions would you like to be available?

• What support could school staff benefit from that is currently unavailable? How do you think this support could be implemented?

• What support could students benefit from that is currently unavailable? How do you think this support could be implemented?

• What support could parents/carers benefit from that is currently unavailable? How do you think this support could be implemented?

### Closure

• Though there were a lot of different opinions it seems that (summarise some of the key points raised).

• Does anybody see the discussion differently? Does anyone want to add or clarify anything?

• Is there any other information regarding your experience of school refusal you think would be useful to share? Are there any other questions? Thanks for coming.
